# Oncogenic Role of Engrailed-2 (*En-2*) in Prostate Cancer Cell Growth and Survival

**Published:** 2008-03-03

**Authors:** Sudeep K. Bose, Rebecca S. Bullard, Carlton D. Donald

**Affiliations:** Department of Pathology and Laboratory Medicine, Medical University of South Carolina (MUSC), 165 Ashley Avenue, PO Box 250620, Charleston, SC—29425, U.S.A

**Keywords:** EN2, PAX2, prostate cancer, oncogene

## Abstract

Prostate cancer is the second leading cause of cancer death among men in the United States of America. However, the molecular mechanisms underlying the disease remain largely unknown. Therefore, the identification of tumor specific molecules that serve as targets for the development of new cancer drugs is considered to be a major goal in cancer research. The mouse Engrailed-2 (*En-2*) gene, which is a homeobox-containing transcription factor was recently identified as a candidate oncogene in breast cancer. Here, we demonstrate that *En-2* is over-expressed in human prostate cancer cells as compared to normal prostate epithelial cells. In addition, our data suggests that *EN2* expression may be positively modulated by *PAX2* transcription factor. Furthermore, down-regulation of *EN2* expression by siRNA resulted in a decrease in *PAX2* expression. We also provide evidence that down-regulation of *EN2* expression causes a dramatic decrease in prostate cancer cell proliferation. Therefore, from our studies we conclude that *En-2* is a candidate oncogene in prostate cancer and its *PAX2*-regulated expression contributes to prostate cancer cell growth.

## Introduction

Prostate cancer is the most common non-cutaneous neoplasm and the second leading cause of male death in the United States (Jemal et al. 2006). The incidence of prostate cancer is known to increase with age. In addition, multiple genetic and epigenetic factors have been implicated in the oncogenesis of prostate cancer, although the molecular mechanisms underlying the disease remain largely unknown (Bostwick and Qian, 1994; Grizzle et al. 1994). Therefore, identification of genetic alterations and genes associated with the development and progression of prostate cancer is important to the understanding of the disease (Kallioniemi and Visakorpi, 1996). Developmental genes that encode transcription factors have an important role in the regulation of specific genes and are necessary for normal growth. In addition, it has been revealed that aberrant expression and structural alteration of transcription factors are often primary molecular mechanisms in tumorigenesis (Rabbitts, 1994).

*PAX* (paired) genes, a family of developmental control genes and transcription factors, regulate tissue development and cellular differentiation in embryos by promoting cell proliferation, migration and survival (Wallin et al. 1998; Buttiglieri et al. 2004; Gnarra and Dressler, 1995). *PAX2* (paired box 2), a class III *PAX* gene, has been shown to be expressed in the developing central nervous system, eye, ear and urogenital tract (Gruss and Walther, 1992; Dressler et al. 1990). Our previous studies demonstrated that *PAX2* expression is an essential requirement for prostate cancer cell survival (Gibson et al. 2007). Furthermore, *PAX* genes have been shown to be capable of acting as proto-oncogenes by transactivating promoters of target genes involved in the regulation of cell growth and apoptosis (Stuart et al. 1995). Therefore, these transcription factors can function both as activators and repressors of transcription.

Another gene class that functions as a homeobox containing transcription factor is the mouse engrailed-2 (*En-2*), which is the murine homologs of the Drosophila segment polarity gene (*En*) (McMahon et al. 1992). It has been observed that *PAX* and *En* genes are the part of genetic networks that control the development of brain and occupy a prominent position in the developmental regulatory hierarchy (Joyner, 1996). Previous reports revealed that *EN2* expression is deregulated in pediatric brain tumor and acute myeloid leukemia (AML) (Kozmik et al. 1995; Nagel et al. 2005). Although *En-2* was recently identified as a candidate oncogene in human breast cancer, very little is known about this regulatory gene relative to organogenesis and cancer (Martin et al. 2005). In the present work, we provide evidence that *EN2* is aberrantly expressed in prostate cancer and is regulated by the *PAX2* transcription factor that promotes prostate cancer cell growth and survival. Furthermore, we found a positive correlation between *En-2* and *PAX2* genes in prostate cancer cell lines, where cells exhibiting decreased *EN2* expression also exhibited a down regulation of *PAX2* expression level.

## Material and Methods

### Cell culture

The prostate cancer cell lines were obtained from the American Type Cell Culture (ATCC). The DU145 were cultured in DMEM medium, PC3 were grown in F-12 medium and LNCaP were grown in RPMI medium (Life Technologies, Inc., Grand Island, NY). Growth media for all three lines was supplemented with 10% (v/v) fetal bovine serum (Life Technologies). The human prostate epithelial cell (hPrEC) cells (Cambrex Bio Science Inc.) were cultured in prostate epithelium basal media supplemented with the SingleQuot^®^ bullet kit (Cambrex Bio Science Inc., Walkersville, MD). All cell lines were maintained at 37 °C and 5% CO_2_.

### siRNA silencing of *PAX2* and *EN2*

Small interfering RNA knock-down was performed as previously described (Gibson et al. 2007). Briefly, a pool of four complementary siRNAs, targeting human PAX2 mRNA (Accession no. NM_003989.1) were synthesized (Dharmacon Research, Lafayette, CO, USA) to knock down expression. To achieve *En-2* gene silencing, siRNA targeting human *EN2* mRNA (Accession no. NM_001427.2) was purchased from Ambion (Applied Biosystem, Inc.). In addition, a second pool of four non-specific siRNAs was used as a negative control (Dharmacon, Inc.). siRNA molecules were transfected with Code-Breaker transfection reagent according to the manufacturer’s protocol (Promega, Inc.).

### RNA isolation and quantitative real-time PCR

RNA was collected after two to four days of siRNA treatment. Approximately 5 × 10^5^ cells were trypsinized and harvested by centrifugation at 4000 × g at 4 °C. Cell pellets were washed twice with ice cold PBS and total RNA was isolated by using the RNAeasy kit (Qiagen Inc., CA, USA). Total RNA (0.5 μg per reaction) was reverse transcribed into cDNA using random primers (Promega, Madison, WI, USA). AMV Reverse Transcriptase II enzyme (500 units per reaction; Promega) was used for first strand synthesis and Tfl DNA Polymerase for second strand synthesis (500 units per reaction; Promega) as per the manufacturer’s protocol. In each case, 50pg of cDNA was used per PCR reaction. Two-step QRT-PCR was performed on cDNA generated using the MultiScribe Reverse Transcriptase from the TaqMan Reverse Transcription System and the SYBR Green PCR Master Mix (Applied Biosystems, Foster City, CA). The primer pair for human *PAX2* (Cat # PPH06881-A) and *En-2* (Cat. # PPH00975A) were purchased from Super Array Bioscience, MD, USA. Forty cycles of PCR were performed under standard conditions at an annealing temperature of 55 °C (ABI Prism 7000). In addition, GAPDH was amplified as a housekeeping gene to normalize the initial content of total cDNA as previously described (Gibson et al. 2007). Relative *PAX2* and *EN2* expression levels were calculated by comparing the prostate cancer cell lines before and after treatment with siRNA to untreated control cells. As a negative control, QRT-PCR reactions without cDNA template were also performed. All reactions were run three times in triplicate.

### Cell proliferation assay

The rate of cell proliferation was determined by [3H] thymidine ribotide ([3H]TdR) incorporation into DNA. Approximately 2.5–5 × 10^4^ cells were plated onto 24-well plates in their appropriate media. Cells were incubated for 72 hours in the absence or presence of siRNA at the indicated concentrations. The cells were exposed to 37 kBq/ ml [methyl-3H] thymidine in the same medium for 6 hours. The adherent cells were fixed by 5% trichloro-acetic acid and lysed in SDS/NaOH lysis buffer overnight. Radioactivity was measured with a Beckman LS3801 liquid scintillation counter. All assays were run three times in triplicate.

### Preparation of total cell extract and western blotting

Cell treated with siRNA or media only were trypsinized, washed with ice-cold PBS and harvested by centrifugation at 100 × g for 5 min at 4 °C. Cell pellets were resuspended in a mammalian lysis buffer containing 1 mM DTT and protease inhibitor cocktail (Sigma Inc., Saint Louis, MI, USA). Lysates were incubated for 15 min on ice followed by centrifugation at 12,000 × g for 10 min at 4 °C to produce total cell extract. Total protein recovered in the supernatant was estimated by Bio-Rad assay and stored in aliquots at −80 °C.

For Western Blot analysis, 18–25 μg of protein sample was mixed with denaturation buffer and boiled for 5 min followed by separation on a 4%–12% NUPAGE Bis-Tris denaturing gel using MES- SDS Runnig buffer. Protein was transferred from the gel to a PVDF membrane that was then blocked for 2–3 hours at room temperature in non-fat dried milk in TBS-T buffer (10 mM Tris/HCl, pH 8.0, 150 mM NaCl and 0.05% Tween-20). Blots were probed with either rabbit anti-PAX2 (Zymed, Inc.) or goat anti-*EN2* antibody (Santa Cruz, Inc.) at 1:1000 dilutions overnight at 4 °C under gentle shaking. The unbound antibodies were removed by 4 × 10 min washes in TBS-T buffer. Next, the membrane was incubated with peroxidase-conjugated secondary antibody (Pierce, IL, USA) for 90 min at room temperature followed by 4 × 10 min washes with TBS-T buffer. Finally, the blot was developed with luminol reagent (Pierce) and visualized by autoradiography. As a negative control, the blots were striped and re-probed for β-actin as a housekeeping gene. Gel quantification was performed using Image J analysis. Each experiment was performed in triplicate.

### Statistical analysis

Statistical analysis was performed using the Student’s t-test for unpaired values. P values were determined by a two-sided calculation, and a P value of less than 0.05 was considered statistically significant. Statistical differences are indicated by asterisks.

## Results

### Analysis of *EN2* expression in prostate cancer cells

**Figure 1:**
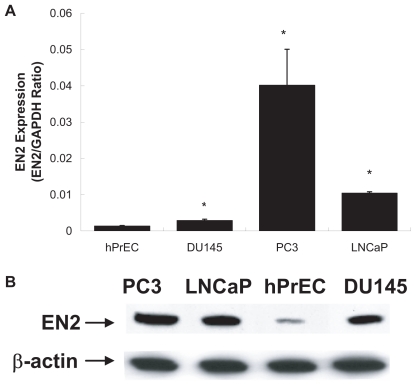
Analysis of *EN2* expression in prostate cells **A**) *EN2* mRNA level were examined by QRT-PCR in hPrEC prostate primary epithelial cells and in DU145, PC3 and LNCaP prostate cancer cells. *EN2* mRNA levels were significantly higher in PC3, LNCaP and DU145 than hPrEC cells. **B**) Western blot analysis of *EN2* was performed on PC3 (lane 1) and LNCaP (lane 2), hPrEC (lane 3) and DU145 (lane 4) cells. *EN2* protein levels was highest in PC3 cells. β-actin was used as an internal control to ensure equal loading.

To investigate *EN2* expression, QRT-PCR was performed on prostate cancer cell lines and hPrEC prostate primary culture. The *EN2* mRNA expression was 2.15-fold higher in DU145, 30-fold higher in PC3 and 7.8-fold higher in LNCaP compared to hPrEC cells (Fig. 1A). *EN2* protein level was examined by Western Blot (Fig. 1B). Our data revealed low levels of *EN2* protein in hPrEC cells (lane 3). However, *EN2* was over-expressed in all of the prostate cancer cell lines. Here *EN2* expression was lowest in DU145, while PC3 cells possessed the greatest amount of expression. *EN2* expression was 8-fold higher in PC3 (lane 1), 6-fold higher in LNCaP (lane 2) and 4-fold higher in DU145 (lane 4) prostate cancer cells compared to hPrEC cells.

### Small interfering RNA-mediated suppression of *EN2*

**Figure 2:**
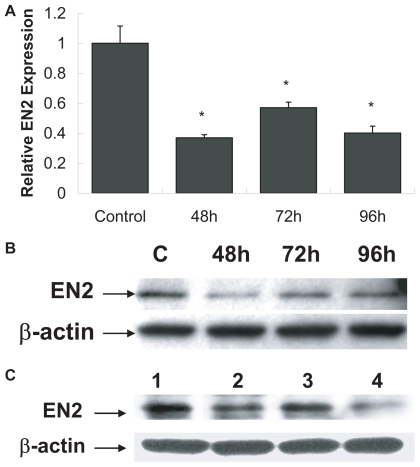
Silencing of *EN2* expression after *En-2* siRNA treatment **A**) QRT analysis of *EN2* mRNA levels reveal a significant decrease in expression during 48–96 hours of *En-2* siRNA treatment as compared to control. **B**) Western blot analysis of *EN2* protein levels in PC3 show a decrease in expression following siRNA treatment during 48 hours to 96 hours of siRNA treatment compared to untreated cells. **C**) Efficiency of *EN2* knock-down showed significant down-regulation of *EN2* after 72 hours of siRNA treatment in PC3 (lane 2) and LNCaP (lane 4) compared to untreated PC3 (lane 1) and LNCaP (lane 3). β-actin was used as an internal control to ensure equal loading.

QRT-PCR analysis of *EN2* expression was monitored in PC3 cells following treatment with *EN2* siRNA. This study revealed a 63% decrease after 48 hours, 43% after 72 hours, and 60% after 96 hours of *En-2* siRNA treatment in PC3 (Fig. 2A). Western Blot analysis was performed to monitor changes in *EN2* protein levels after selective targeting and inhibition by *En-2* specific siRNA in PC3 prostate cancer cells. Following treatment, protein expression decreased by 70% at 48 hours, 20% at 72 hours and 26% at 96 hours (Fig. 2B). Efficiency of *EN2* knock-down was compared in PC3 and LNCaP cell lines (Fig. 2C). After siRNA treatment for 72 hours, *EN2* protein levels decreased by 25% in PC3 (lane 2), and by 60% in LNCaP (lane 4) when compared to untreated PC3 (lane 1) and LNCaP (lane 3) cells.

### Effect of *EN2* knockdown on prostate cancer cell growth

**Figure 3:**
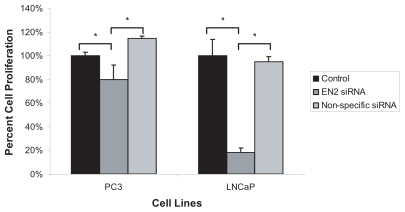
Analysis of prostate cancer cell growth after *EN2* siRNA treatment PC3 and LNCaP prostate cancer cells were treated with 150 nM of *En-2* siRNA or non-specific negative control siRNAs for 3 days after which cell proliferation was examined by thymidine incorporation assay. Cell proliferation was decreased by 20% in PC3 and 80% in LNCaP cells.

To examine the effect of therapeutic targeting and inhibition of *EN2* expression on the rate of prostate cancer cell growth, cell proliferation was monitored by a thymidine incorporation assay after 72 hours of siRNA treatment against *En-2* in PC3 and LNCaP cells. Treatment of PC3 cells with 150 nM *EN2* siRNA resulted in a 20% inhibition in cell proliferation rate compared to cell treated with media only (Fig. 3). However, treatment of LNCaP cells with *En-2* siRNA resulted in an 81% decrease in proliferation rate as compared to those treated with the non-specific siRNA. As a negative control, cells were treated with an equal amount of non-specific siRNA, and there was no significant change in cell viability.

### Effect of PAX2 knockdown on *EN2* expression in prostate cancer

**Figure 4:**
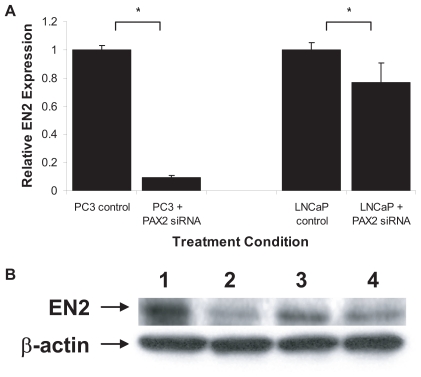
Analysis of *EN2* expression after PAX2 siRNA treatment **A**) *EN2* mRNA expression was examined after 72 hours of PAX2 siRNA treatment of PC3, as well as, LNCaP prostate cancer cell lines by QRT-PCR. Treatment with PAX2 siRNA resulted in a dramatic decrease in *EN2* expression in PC3 cells and a modest decrease in LNCaP. **B**) Western blot analysis of *EN2* protein expression revealed a 70% decrease in protein levels in PC3 after 72 hours PAX2 siRNA treatment (lane 2) compared to untreated PC3 cells (lane 1). In LNCaP there was a 26% decrease in *EN2* protein levels after *PAX2* siRNA (lane 4) compared to untreated control cells (lane 3). β-actin was used as an internal control to ensure equal loading.

To determine the role of *PAX2* on *EN2* expression in prostate cancer, PC3 and LNCaP cells were treated for 3 days with a pool of siRNA specifically targeted against *PAX2*. We previously demonstrated that siRNA knockdown of *PAX2* expression occurs as early as 2 days in the prostate cancer cell lines (Gibson et al. 2007). QRT-PCR analysis revealed that *EN2* mRNA level was down-regulated in PC3 cell line by 91% as compared to control cells treated with media only (Fig. 4A). In addition, *EN2* mRNA in LNCaP cells was suppressed by 23% compared to control. Western blot analysis of *EN2* protein expression in the prostate cancer cell lines after 3 days of *PAX2* siRNA treatment (Fig. 4B) demonstrated that *EN2* expression was decreased 70% in PC3 (lane 2) and 26% in LNCaP (lane 4) prostate cancer cell lines as compared to PC3 (lanes 1) and LNCaP (lanes 3) controls.

### Analysis of *PAX2* expression after *EN2* knockdown in prostate cancer

**Figure 5:**
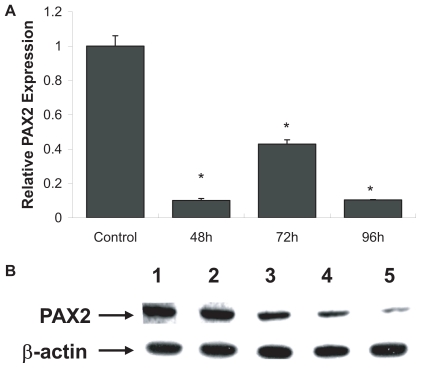
Analysis of *PAX2* expression after *EN2* knockdown in prostate cancer cells **A**) QRT-PCR was performed to examine *PAX2* mRNA levels in the LNCaP prostate cancer cell line after *En-2* siRNA treatment. Following treatment with *En-2* siRNA, *PAX2* expression levels decreased by 60–90% during 48–96 hours. Data is presented as relative expression compared to untreated LNCaP cells. **B**) Examination of *PAX2* protein levels in LNCaP after *En-2* siRNA treatment by Western blot analysis revealed a significant decrease during 48 hours to 96 hours (lanes 3–5) compared to untreated cells (lane 1). As a negative control, *PAX2* expression was also tested in presence of non-specific siRNA (lane 2). β-actin was used as an internal control to ensure equal loading.

QRT-PCR analysis of *PAX2* was performed in LNCaP cells after treatment with *EN2* siRNA to determine whether *En-2* can modulate *PAX2* expression in prostate cancer. Our data revealed that *PAX2* mRNA level was significantly decreased by 90% at 48 hours, 67% at 72 hours and 90% at 96 hours in LNCaP cells (Fig. 5A). Furthermore, to test the correlation between *PAX2* and *EN2* at the protein level, Western blot analysis was performed. Here, *PAX2* protein level decreased by 50% at 48 hours (lane 3), by 66% at 72 hours (lane 4) and by 72% at 96 hours (lane 5) after *En-2* siRNA treatment compared to untreated cells (lane 1) and non-specific siRNA treated cells (lane 2) (Fig. 5B).

## Discussion

There is considerable heterogeneity in the biological aggressiveness of prostate cancer. Therefore, the identification of reliable diagnostic and/or prognostic markers and the development of novel mechanism-based therapeutic treatment regimens are urgently needed (Tang and Porter, 1997). It has been reported that the relationship between developmental processes and oncogenesis involves deregulated cell growth (Kallioniemi and Visakorpi, 1996). The *En-1* and *En-2* genes, homologues of the mouse and drosophila segmentation gene engrailed (*En*), encode homeodomain transcription factors (Joyner, 1996). Little is known about the role of engrailed genes in tumorogenesis. In 2005, Martin et al. provided the first evidence of *En-2* as a candidate oncogene, which is aberrantly expressed in breast cancer and has a role in mammary tumorigenesis. *EN2* was also detected in SAGE libraries derived from human brain glioblastoma, colon and ovarian carcinomas (Martin et al. 2005). Here we demonstrated that *EN2* is over-expressed in human prostate cancer cells compared to normal prostate epithelial cells (Fig. 1). Therefore, our data suggests that *EN2* is over-expressed in prostate cancer and may contribute to prostate tumorigenesis.

In this study we examined the role of EN2 over-expression in the following prostate cancer cell lines: LNCaP, which is p53 wild-type and androgen receptor (AR) positive; DU145, which has a mutated p53 and is AR negative; and the p53-null line PC3, which is also AR negative. *EN2* expression was highest in PC3 cells compared to the other two lines with the greatest difference existing between PC3 and LNCaP. This finding was particularly interesting given that LNCaP cells are relatively slow-growing and are thought to represent early-stage prostate cancer, while PC3 cells are fast-growing and are thought to represent late-stage, aggressive prostate cancer. Previous studies have demonstrated that the suppression of *EN2* in breast cancer cell lines by siRNA against *En-2* resulted in a significant decrease in their proliferation rate (Martin et al. 2005). Here we found a similar response in prostate cancer cells (Fig. 3). These findings are in line with our previous observation that siRNA knockdown of *PAX2* resulted in dramatic decreases in prostate cancer cell growth (Gibson et al. 2007). In addition, PAX2 expression levels were found to be significantly higher in PC3 cell line compared to LNCaP cells. Given that suppression of *EN2* results in the down-regulation of *PAX2* expression, this observation may be due to the reactivation of anti-proliferative factors that are negatively regulated by PAX2 such as p53. This further implicates *En-2* as a therapeutic target for cancer either directly or indirectly via targeting *PAX2*.

To date, little is known about the factors that regulate *EN2* expression. Studies in drosophila, zebra fish and mice indicate that the *PAX*-*En* genetic pathway is conserved during evolution and they interact with each other (Song et al. 1996). *PAX2*, a member of the *PAX* gene family of transcriptional regulators, is essential during early development of the urogenital system (Eccles et al. 2002). In addition, *PAX2* has been shown to be aberrantly over-expressed in urogenital cancers, including prostate carcinomas (Discenza et al. 2003; Khoubehi et al. 2001; Muratovska et al. 2003). Studies have shown that *PAX2* expression in malignant cells generates a proliferation stimulus, that may be an integral part of the multi-step oncogenic transformation process (Gibson et al. 2007). *PAX2* contains novel recognition sequences within the paired domain and activating domains within the C-terminal region that facilitate repression and activation of gene transcription through DNA binding (Havick et al. 1999). PAX2 has also been shown to interact with the tumor suppressor genes WT1 and p53 (Stuart et al. 1995; Dehbi et al. 1996).

Here we have demonstrated that suppression of *PAX2* expression by siRNA resulted in a decrease in *En-2* gene expression. These findings suggest that *PAX2* may be a transcriptional activator of *En-2* although this was not examined in this study. Furthermore, we found a positive correlation between *EN2* and *PAX2* where prostate cancer cells exhibiting decreased *EN2* expression also possessed decreased *PAX2* levels. This indicates that *EN2* expression may influence *PAX2* through a feedback mechanism. Taken together, it is plausible that deregulated expression of *PAX2* and *EN2* may ultimately promote tumor progression specifically via cancer cell proliferation and survival.

Although there have been significant advances made in cancer therapeutics, little progress has been made in the treatment of the advanced stage of cancers. Successful drug treatment of prostate cancer requires the use of therapeutics with specific effects on target cells with overall aim of inducing apoptosis while maintaining minimal clinical effects on the host. Here we demonstrate that *En-2* may contribute to the process of oncogenesis by conferring a growth advantage to prostate cancer cells by supporting cellular proliferation. Collectively, our data suggest that *En-2* may prove useful as a specific biomarker marker of prostate malignancy and further investigation may explain its mechanistic contribution to the tumorigenic process.

## Abbreviations

En-2: engrailed-2 gene
EN2: engrailed-2 protein
siRNA: small interfering RNA
PBS: Phosphate Buffer Saline
DTT: Dithiothreitol
TBS-T: Tris Buffer Saline- Tween-20
hPrEC: human prostate epithelial cell
C: control
T: treated
